# The impact of body mass index on prognosis in patients with colon carcinoma

**DOI:** 10.1007/s00384-022-04146-2

**Published:** 2022-04-14

**Authors:** Julian Fuchs, Vera S Schellerer, Maximilian Brunner, Carol I Geppert, Robert Grützmann, Klaus Weber, Susanne Merkel

**Affiliations:** 1 Department of Surgery, Friedrich-Alexander-Universität Erlangen-Nürnberg, Krankenhausstr. 12, 91054 Erlangen, Germany; 2 Department of Surgery, Moritz-Arndt-Universität Greifswald, Greifswald, Germany; 3 Institute of Pathology, Friedrich-Alexander-Universität Erlangen-Nürnberg, Erlangen, Germany; 4 Comprehensive Cancer Center Erlangen–European Metropolitan Area of Nuremberg (CCC ER-EMN), Erlangen, Germany

**Keywords:** Colon carcinoma, Body mass index, Obesity paradox, Prognosis, Overall survival

## Abstract

**Background:**

The impact of body mass index (BMI) on prognosis in patients with curatively resected stage I–III colon carcinoma was analyzed.

**Methods:**

The prospectively collected data of 694 patients who underwent complete mesocolic excision between 2003 and 2014 were analyzed. BMI was classified into four categories: underweight (BMI < 18.5 kg/m^2^; *n* = 13), normal weight (BMI 18.5 to 24.9 kg/m^2^; *n* = 221), overweight (BMI 25.0 to 29.9 kg/m^2^; *n* = 309), and obese (BMI ≥ 30.0 kg/m^2^; *n* = 151). Univariate and multivariate analyses for comparison of prognosis were performed.

**Results:**

The 5-year rate of locoregional recurrence in all 694 patients was 2.1%, and no differences were found with respect to BMI (*p* = 0.759). For distant metastasis, the 5-year rate for all patients was 13.4%, and BMI did not have a significant impact (*p* = 0.593). The 5-year rate of disease-free survival for all 694 patients was 72.4%. The differences with respect to BMI were not found to be significant in univariate analysis (*p* = 0.222). In multivariate Cox regression analysis, disease-free survival was significantly better in obese patients (HR 0.7; *p* = 0.034). Regarding overall survival, the 5-year rate for all patients was 78.1%. In univariate analyses, no significant differences were found for BMI (*p* = 0.094). In the Cox regression analysis, overweight and obese patients had significantly better survival (overweight: HR 0.7; *p* = 0.027; obese: HR 0.6; *p* = 0.019).

**Conclusion:**

The better survival of overweight and obese patients in multivariate analyses must be interpreted with caution. It is influenced by several factors and seems to correspond to the phenomenon of the obesity paradox.

## Introduction

The World Health Organization’s (WHO) body mass index (BMI) is a common anthropometric tool to roughly estimate a person’s nutritional status. BMI is widely available. This is the strength of this measure. It is not associated with high costs and only requires patient data (weight and height) that are usually collected in everyday clinical practice [1]. Although BMI cannot provide precise individual information about body fat content or identify whether body fat is visceral or subcutaneous, it has been shown that BMI correlates with direct body fat measurements at the group level [2].

In recent decades, the average BMI of the world population has steadily increased. In 1975, 40.0% of Europe’s population was found to be at least overweight (BMI ≥ 25.0 kg/m^2^); in 2016, 62.3%. In addition, the rate of obesity (BMI ≥ 30.0 kg/m^2^) rose from 10.3% in 1975 to 25.3% in 2016 [3].

Obesity is considered to be a risk factor for many chronic diseases. These include cardiovascular disease [4], type 2 diabetes [5], metabolic syndrome [6], chronic kidney disease [7], chronic liver disease [8], and various types of cancer. Colon cancer is considered to be one of these types [2, 9–11].

It is important to know the body fat measures before major surgery to estimate problems with anesthesia. Obese patients have a higher risk for cardiovascular or pulmonary intraoperative and postoperative problems, longer operation time, and longer hospital stay due to increased risk of infection and poor wound healing. However, in colon cancer, not only the short-course but also the long-term prognosis is discussed controversially in the context of obesity. This study’s aim was to investigate the association of BMI and long-term prognosis in patients with colon cancer after complete mesocolic excision (CME). The prognostic importance of BMI will be discussed in the context of other relevant patient- and tumor-related prognostic factors, and the obesity paradox will be addressed.

## Methods

Between 1 January 2003 and 31 December 2014, 1172 patients with primary colon carcinoma (invasion at least into the submucosa) with a distal margin more than 16 cm from the anal verge were treated at the Department of Surgery, University Hospital Erlangen, Germany. Data were prospectively collected in the Erlangen Registry for Colorectal Carcinomas (ERCRC).

Patients with the following criteria were excluded: multiple colon carcinomas (*n* = 53), other previous or synchronous malignancies (*n* = 166), carcinomas related to familial polyposis, ulcerative colitis, or Crohn’s disease (*n* = 22), distant metastasis (*n* = 222), neoadjuvant treatment (*n* = 5), and appendiceal carcinoma (*n* = 3). In addition, patients with unknown tumor status (*n* = 4) and noncurative resection (*n* = 3) were excluded, as were one patient with R1 resection (normal weight), one patient with R2 resection (normal weight), and one patient with unknown R status (RX, class I obesity). In total, 694 patients were included for the statistical analysis.

The WHO BMI categories were applied for weight classification. BMI was defined as weight in kilograms divided by the square of height in meters (kg/m^2^). The routinely collected data of all patients’ weight and height, measured during medical examinations in the hospital before surgery, were used. BMI classifies the categories of underweight (BMI < 18.5 kg/m^2^), normal weight (BMI 18.5 to 24.9 kg/m^2^), overweight (BMI 25.0 to 29.9 kg/m^2^), class I obesity (BMI 30.0 to 34.9 kg/m^2^), class II obesity (BMI 35.0 to 39.9 kg/m^2^), and class III obesity (BMI ≥ 40 kg/m^2^). For statistical analyses, we summarized classes I to III as obese (BMI ≥ 30.0 kg/m^2^) [12].

All patients underwent surgery with complete mesocolic excision (CME). The aim of this standardized method is to separate the mesocolic from the parietal fascia. Furthermore, the supplying arteries were ligated at their origin to achieve maximal lymph node harvest [13, 14]. The extent of resection depended on the tumor location. Hemicolectomy, left hemicolectomy, and sigmoid resection were summarized as standard resection, and extended right and left hemicolectomy as well as subtotal and total colectomy were summarized as extended resection. The right colon includes the cecum, ascending colon, right flexure, and right two-thirds of the transverse colon, whereas the left one-third of the transverse colon, the left flexure, the descending colon, and the sigmoid colon comprise the left colon. Emergency surgery was defined as surgery required within 48 h after admission [15].

The American Society of Anesthesiologists’ (ASA) physical status classification system was used, with the following risk classes: class I = “a healthy person”; class II = “a patient with mild systemic disease”; class III = “a patient with severe systemic disease”; class IV = “a patient with severe systemic disease that is a constant threat to life” [16].

The anatomical extent of the tumors was categorized according to the TNM classification of the UICC. The detailed documentation allowed a classification of all carcinomas according to the current eighth edition [17].

Intraoperative local tumor cell dissemination was defined as iatrogenic disruption of the tumor during mobilization and/or incision into the tumor tissue. Patients with stage III disease received adjuvant chemotherapy after curative (R0) resection. Clinically evident anastomotic leaks were classified according to Rahbari and colleagues [18]. Only grade B or grade C leakages were considered for statistical analysis.

All patients were followed up until 1 January 2018 or death. Patients routinely received follow-up according to the German S3 Guidelines for Colorectal Cancer for 5 years. The scheduled follow-up included detailed anamnesis, physical examination, analyses of carcinoembryonic antigen (CEA) levels, abdominal ultrasonography, and colonoscopy [19]. Follow-up was carried out every 6 months in the first 2 years and afterwards on a yearly basis. After 5 years of regular follow-up, at least information about the patients’ vital status from the national registration office was collected yearly.

### Statistical analyses

The *χ*^2^ test and Fisher’s exact test were used to compare frequencies, and the Mann–Whitney *U* test was used for continuous data. For estimation of overall survival, disease-free survival, rates of locoregional recurrence, and distant metastasis, we used the Kaplan–Meier method. The endpoint of disease-free survival was recurrence (locoregional or distant) or death from any cause. The endpoint of overall survival was death from any cause. The log-rank test was used to compare survival curves. Age, sex, BMI, ASA, tumor location, emergency surgery, surgical intervention, and tumor stage were initially analyzed univariately with regard to locoregional recurrence, distant metastases, disease-free survival, and overall survival. In addition to BMI, all significant factors in univariate analysis (*p* < 0.05) were included in a multivariate Cox regression model to identify independent risk factors. For BMI, normal weight was the reference variable. *p* < 0.05 was considered significant. The statistical software package SPSS version 24 (IBM, Armonk, New York, USA) was used for all analyses.

## Results

A total of 694 patients with colon carcinoma were analyzed. The median follow-up was 81 months (IQR 48–122); at the date of analysis (January 01, 2018), 243 (35.0%) patients had died. Table 1 shows the patient and tumor characteristics. The median age was 67 years (range: 17–93 years). According to the WHO BMI classification, there were 13 underweight (1.9%), 221 normal weight (31.8%), 309 overweight (44.5%), and 151 obese (21.8%) patients. The patient and tumor characteristics with respect to BMI classification are shown in Table 2. Males were found to have significantly higher rates of overweight and obesity than females (*p* < 0.001).

**Table 1:** Patient and tumor characteristics, *n* = 694

		*n*	%
Age median (range) (years)		67 (17–93)	
Sex	Male	405	58.4
	Female	289	41.6
BMI (kg/m^2^)	Underweight < 18.5	13	1.9
	Normal weight 18.5–24.9	221	31.8
	Overweight 25.0–29.9	309	44.5
	Class I obesity 30.0–34.9	112	16.1
	Class II obesity 35.0–39.9	33	4.8
	Class III obesity ≥ 40.0	6	0.9
ASA*	ASA 1–2	504	74.0
	ASA 3–4	177	26.0
CEA**	Normal (< 5 ng/ml)	453	81.3
	Elevated (≥ 5 ng/ml)	104	18.7
Histological type	Adenocarcinoma (8140)	629	90.6
	Other types (8480, 8490, 8510)	65	9.4
Tumor location	Right colon	318	45.8
	Left colon	376	54.2
Emergency surgery	No, elective surgery	630	90.8
	Yes, emergency surgery	64	9.2
Surgical procedure	Standard resection	556	80.1
	Extended resection	138	19.9
pT category	pT1	96	13.8
	pT2	151	21.8
	pT3	380	54.8
	pT4	67	9.7
pN category	pN0	473	68.2
	pN1	163	23.5
	pN2	58	8.4
Stage (UICC)	Stage I	202	29.1
	Stage II	271	39.0
	Stage III	221	31.8

*BMI* body mass index, *ASA* American Society of Anesthesiologists Classification, *CEA* carcinoembryonic antigen

^*^ASA missing in 13 patients; **CEA missing in 137 patients

**Table 2:** BMI with respect to patient and tumor characteristics

BMI (kg/m^2^)	Underweight < 18.5	Normal weight 18.5–24.9	Overweight 25.0–29.9	Obese ≥ 30.0	*p*
	(*n* = 13)	(*n* = 221)	(*n* = 309)	(*n* = 151)	
	*n* (%)	*n* (%)	*n* (%)	*n* (%)	
Age median (range) (years)	68 (45–85)	67 (17–93)	68 (38–92)	66 (27–91)	0.565
Sex					
Male	1 (8)	109 (49.3)	201 (65.0)	94 (62.3)	
Female	12 (93)	112 (50.7)	108 (35.0)	57 (37.7)	< 0.001
ASA*					
ASA 1–2	11 (92)	167 (76.6)	226 (75.1)	100 (66.7)	
ASA 3–4	1 (9)	51 (23.4)	75 (24.9)	50 (33.3)	0.069
Histological type					
Adenocarcinoma (8140)	10 (77)	196 (88.7)	285 (92.2)	138 (91.4)	
Others (8480, 8490, 8510)	3 (23)	25 (11.3)	24 (7.8)	13 (8.6)	0.179
Tumor location					
Right colon	7 (54)	100 (45.2)	144 (46.6)	67 (44.4)	
Left colon	6 (47)	121 (54.8)	165 (53.4)	84 (55.6)	0.903
Emergency surgery					
No, elective surgery	12 (93)	195 (88.2)	279 (90.3)	144 (95.4)	
Yes, emergency surgery	1 (8)	26 (11.8)	30 (9.7)	7 (4.6)	0.131
Surgical procedure					
Standard resection	10 (77)	163 (73.8)	256 (82.8)	126 (83.4)	
Extended resection	3 (24)	58 (26.2)	53 (17.2)	25 (16.6)	0.043
pT category					
pT1	5 (38)	28 (12.7)	44 (14.2)	19 (12.6)	
pT2	2 (15)	50 (22.6)	66 (21.4)	33 (21.9)	
pT3	5 (38)	125 (56.6)	160 (51.8)	90 (59.6)	
pT4	1 (8)	19 (8.6)	39 (12.6)	9 (6.0)	0.126
pN category					
pN0	13 (100)	160 (72.4)	205 (66.3)	95 (62.9)	
pN1	0	42 (19.0)	81 (26.2)	40 (26.5)	
pN2	0	19 (8.6)	23 (7.4)	16 (10.6)	0.060
Stage (UICC)					
Stage I	7 (54)	70 (31.7)	86 (27.8)	39 (25.8)	
Stage II	6 (47)	90 (40.7)	119 (38.5)	56 (37.1)	
Stage III	0 (0)	61 (27.6)	104 (33.7)	56 (37.1)	0.075

*BMI* body mass index, *ASA* American Society of Anesthesiologists Classification

^*^ASA missing in 13 patients

### Indicators of process quality

The indicators of process quality (Table 3) showed no significant difference between the weight classes with respect to the number of regional lymph nodes examined (≥ 12 lymph nodes in 98.4%), intraoperative local tumor dissemination (0.4%), adjuvant chemotherapy in stage III (78.6%), postoperative morbidity (21.6%), and postoperative mortality (in-hospital mortality, 2.4%).

**Table 3:** Indicators of process quality

BMI (kg/m^2^)	All	Underweight < 18.5	Normal weight 18.5–24.9	Overweight 25.0–29.9	Obese ≥ 30.0	*p*
	(*n* = 694)	(*n* = 13)	(*n* = 221)	(*n* = 309)	(*n* = 151)	
Number of l.n. examined (a) median (range)	29 (1–145)	29 (14–51)	30 (11–145)	28 (4–101)	28 (1–72)	0.936
(b) ≥ 12 l.n. examined	683/694 (98.4%)	13/13 (100%)	219/221 (99.1%)	303/309 (98.1%)	148/151 (98.0%)	0.737
Number of l.n. examined in pN0 standard resection (a) median (range)	26 (1–92)	27 (14–45)	26 (11–84)	26 (4–92)	26 (1–68)	0.811
(b) ≥ 12 l.n. examined	367/376 (97.6%)	10/10 (100%)	118/120 (98.3%)	164/169 (97.0%)	75/77 (97.4%)	0.859
Intraoperative local tumor cell dissemination	3/690 (0.4%)	0/13 (0%)	0/218 (0%)	3/309 (1.0%)	0/150 (0%)	0.685
Adjuvant chemotherapy in stage III	165/210 (78.6%)	-	41/56 (73.2%)	83/100 (83.0%)	41/54 (75.9%)	0.310
Postoperative morbidity	150/694 (21.6%)	2/13 (15.4%)	58/221 (26.2%)	55/309 (17.8%)	35/151 (23.2%)	0.113
Anastomotic leak	22/679 (3.2%)	0/13 (0%)	6/213 (2.8%)	7/304 (2.3%)	9/149 (6.0%)	0.162
In-hospital mortality	17/694 (2.4%)	0/13 (0%)	10/221 (4.5%)	4/309 (1.3%)	3/151 (2.0%)	0.104
30-day mortality	16/694 (2.3%)	0/13 (0%)	9/221 (4.1%)	4/309 (1.3%)	3/151 (2.0%)	0.184
90-day mortality	24/694 (3.5%)	0/13 (0%)	11/221 (5.0%)	8/309 (2.6%)	5/151 (3.3%)	0.440

*l.n.* lymph nodes

### Indicators of outcome quality

#### Locoregional recurrences

**Fig. 1:**
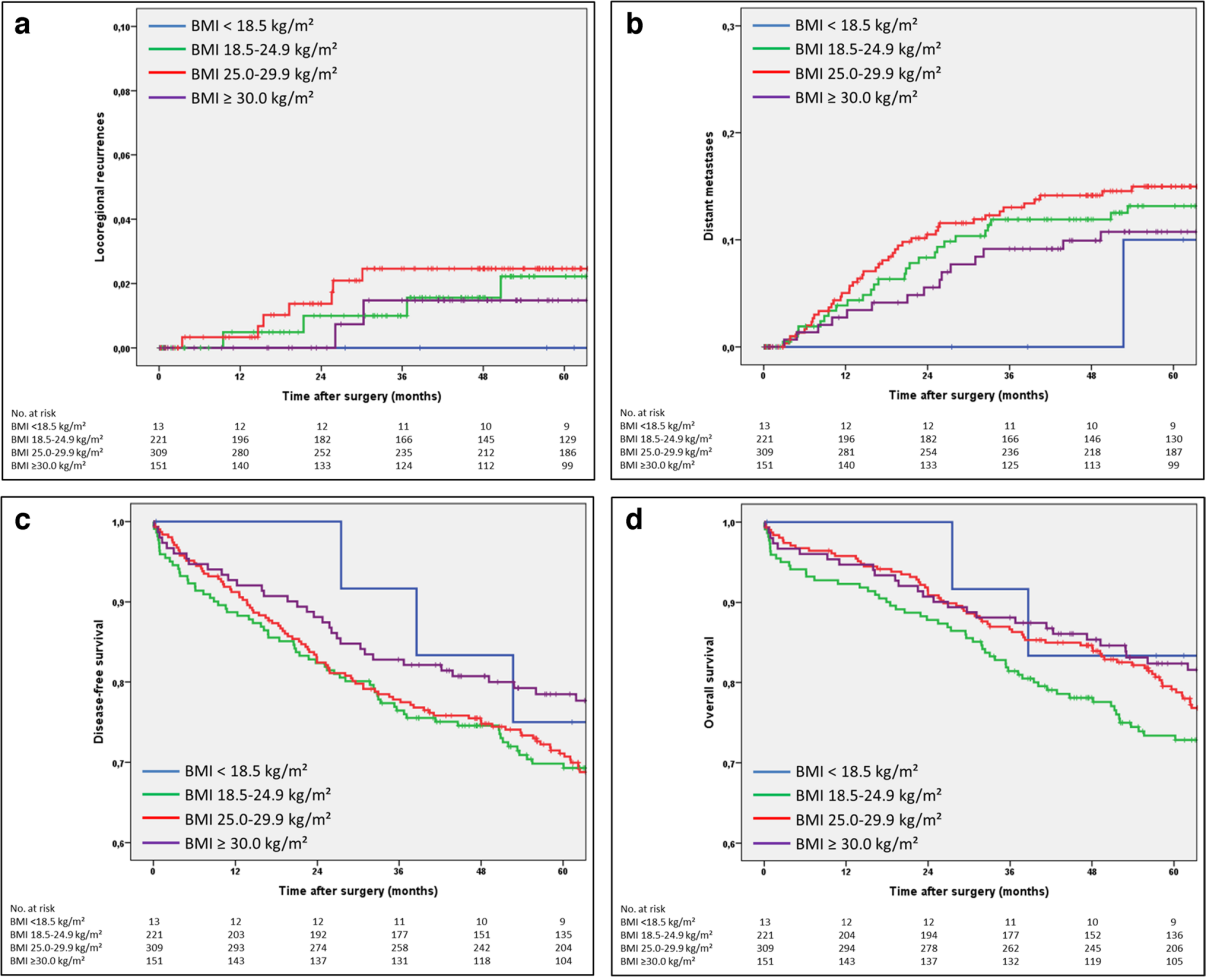
**a** Kaplan–Meier curves of the time to locoregional recurrence (*n* = 694; *p* = 0.759). **b** Kaplan–Meier curves of the time to distant metastases (*n* = 694; *p* = 0.593). **c** Kaplan–Meier curves of disease-free survival (*n* = 694; *p* = 0.222). **d** Kaplan–Meier curves of overall survival (*n* = 694; *p* = 0.094)

The 5-year rate of locoregional recurrence in all 694 patients was 2.1% (95% CI 0.9–3.3). No significant differences were found with respect to age, sex, tumor location, emergency presentation, surgical procedure, tumor stage, or BMI (*p* = 0.759; Fig. 1a).

#### Distant metastases

The 5-year rate of distant metastases (Table 4) for all 694 patients was 13.4% (95% CI 10.7–16.1). Age, sex, tumor location, type of surgery, and ASA grade did not have a significant impact on the rate of distant metastases, nor did BMI (*p* = 0.593; Fig. 1b). Emergency surgery (5-year rate 24.5% vs. 12.4%) and tumor stage (stage I, 3.8%; stage II, 12.2%; stage III, 24.1%) were found to be significant prognostic factors for distant metastasis, which was confirmed in multivariate Cox regression analysis. BMI class had no influence on distant metastasis.

**Table 4:** Distant metastases, *n* = 694

		Univariate analysis			Multivariate analysis adjusted for age		
	*n*	5-year rate	95% CI	*p*	Hazard ratio	95% CI	*p*
All	694	13.4%	10.7–16.1				
Age							
≤ 65	289	14.4%	10.3–18.5				
> 65	405	12.6%	9.1–16.1	0.526			
Sex							
Male	405	14.6%	11.1–18.1				
Female	289	11.7%	7.8–15.6	0.397			
BMI (kg/m^2^)							
Underweight < 18.5	13	10.0%	0–28.6		0.8	0.1–6.3	0.871
Normal weight 18.5–24.9	221	11.9%	7.4–16.4		1.0		
Overweight 25.0–29.9	309	15.0%	10.9–19.1		1.0	0.6–1.6	0.925
Obese ≥ 30.0	151	10.7%	5.6–15.8	0.593	0.7	0.4–1.3	0.218
ASA*							
ASA 1–2	504	13.0%	10.1–15.9				
ASA 3–4	177	14.4%	8.7–20.1	0.444			
Tumor location							
Right colon	318	12.9%	9.0–16.8				
Left colon	376	13.8%	10.1–17.5	0.830			
Emergency surgery							
No, elective surgery	630	12.4%	9.7–15.1		1.0		
Yes, emergency surgery	64	24.5%	12.9–36.1	0.004	2.1	1.2–3.7	0.012
Surgical procedure							
Standard resection	555	13.0%	10.1–15.9				
Extended resection	139	15.2%	8.9–21.5	0.532			
Stage (UICC)							
Stage I	202	3.8%	1.1–6.5		1.0		
Stage II	271	12.2%	8.1–16.3		2.5	1.2–5.0	0.014
Stage III	221	24.1%	18.2–30.0	< 0.001	5.4	2.7–10.6	< 0.001

*BMI* body mass index, *ASA* American Society of Anesthesiologists Classification

^*^ASA missing in 13 patients

#### Disease-free survival

The 5-year rate of disease-free survival (Table 5) for all 694 patients was 72.4% (95% CI 69.1–75.7). There was significantly better disease-free survival in patients who were younger, in patients with ASA performance status 1–2, in patients with elective surgery, in patients with standard resections, and in patients with left-sided colon tumors and in stage I–II tumors. The differences with respect to BMI were not found to be significant in univariate analysis (*p* = 0.222). In multivariate Cox regression analysis, emergency surgery, stage III, and ASA 3–4 were found to be independent prognostic factors for worse disease-free survival. With respect to BMI, disease-free survival was significantly better in obese patients (hazard ratio 0.7; 95% confidence interval 0.5–1.0; *p* = 0.034).

**Table 5:** Disease-free survival, *n*  = 694

		Univariate analysis			Multivariate analysis adjusted for age		
	n	5-year rate	95% CI	p	Hazard ratio	95% CI	p
All	694	72.4%	69.1–75.7				
Age							
≤ 65	289	79.8%	75.1–84.5				
> 65	405	67.1%	62.4–71.8	< 0.001			
Sex							
Male	405	69.6%	65.1–74.1				
Female	289	76.2%	71.3–81.1	0.200			
BMI (kg/m^2^)							
Underweight < 18.5	13	75.0%	50.5–99.5		1.2	0.4–3.3	0.702
Normal weight 18.5–24.9	221	69.8%	63.7–75.9		1.0		
Overweight 25.0–29.9	309	71.1%	66.0–76.2		0.8	0.6–1.1	0.202
Obese ≥ 30.0	151	78.5%	71.8–85.2	0.222	0.7	0.5–1.0	0.034
ASA*							
ASA 1–2	504	78.1%	74.4–81.8		1.0		
ASA 3–4	177	55.8%	48.4–63.2	< 0.001	1.8	1.4–2.3	< 0.001
Tumor location							
Right colon	318	69.9%	64.8–75.0		1.0		
Left colon	376	74.4%	69.9–78.9	0.014	0.8	0.6–1.0	0.071
Emergency surgery							
No, elective surgery	630	74.9%	71.4–78.4		1.0		
Yes, emergency surgery	64	46.8%	34.3–59.3	< 0.001	1.7	1.2–2.4	0.005
Surgical procedure							
Standard resection	555	73.2%	69.5–76.9		1.0		
Extended resection	139	68.8%	61.0–76.6	0.025	1.1	0.8–1.4	0.630
Stage (UICC)							
Stage I	202	83.4%	78.1–88.7		1.0		
Stage II	271	71.7%	66.2–77.2		1.2	0.9–1.6	0.278
Stage III	221	63.1%	56.6–69.6	0.001	1.7	1.2–2.3	0.002

*BMI* body mass index, *ASA* American Society of Anesthesiologists Classification

^*^ASA missing in 13 patients

#### Overall survival

The 5-year overall survival rate (Table 6) for all 694 patients was 78.1% (95% CI 75.0–81.2). Significant differences were found in univariate analyses with respect to age, ASA, tumor location, emergency presentation, surgical procedure, and stage but not for BMI (*p* = 0.094). In the Cox regression analysis, ASA, tumor location, and stage III were identified as independent prognostic factors. In addition, overweight and obese patients had significantly better survival in multivariate analysis (overweight: HR 0.7; 95% CI 0.5–1.0; *p* = 0.027; obese: HR 0.6; 95% CI 0.4–0.9; *p* = 0.019).

**Table 6:** Overall survival, *n* = 694

		Univariate analysis			Multivariate analysis adjusted for age		
	n	5-year rate	95% CI	p	Hazard ratio	95% CI	p
All	694	78.1%	75.0–81.2				
Age							
≤ 65	289	88.1%	84.2–92.0				
> 65	405	71.0%	66.5–75.5	< 0.001			
Sex							
Male	405	75.5%	71.2–79.8				
Female	289	81.8%	77.3–86.3	0.268			
BMI (kg/m^2^)							
Underweight < 18.5	13	83.3%	62.1–100		1.2	0.4–3.7	0.809
Normal weight 18.5–24.9	221	73.4%	67.5–79.3		1.0		
Overweight 25.0–29.9	309	79.2%	74.5–83.9		0.7	0.5–1.0	0.027
Obese ≥ 30.0	151	82.4%	76.3–88.5	0.094	0.6	0.4–0.9	0.019
ASA*							
ASA 1–2	504	85.0%	81.9–88.1		1.0		
ASA 3–4	177	58.5%	51.1–65.9	< 0.001	2.0	1.5–2.7	< 0.001
Tumor location							
Right colon	318	73.9%	69.0–78.8		1.0		
Left colon	376	81.6%	77.7–85.5	0.001	0.7	0.6–0.9	0.018
Emergency surgery							
No, elective surgery	630	80.3%	77.2–83.4		1.0		
Yes, emergency surgery	64	56.2%	43.7–68.7	< 0.001	1.4	1.0–2.1	0.070
Surgical procedure							
Standard resection	555	79.4%	75.9–82.9		1.0		
Extended resection	139	73.2%	65.8–80.6	0.016	1.1	0.8–1.5	0.589
Stage (UICC)							
Stage I	202	85.9%	81.0–90.8		1.0		
Stage II	271	78.0%	72.9–83.1		1.2	0.8–1.6	0.373
Stage III	221	71.2%	65.1–77.3	0.023	1.5	1.0–2.1	0.026

*BMI* body mass index, *ASA* American Society of Anesthesiologists Classification

^*^ASA missing in 13 patients

In summary, overweight and obese BMI were found to be independent favorable prognostic factors for overall and disease-free survival but not for distant metastasis.

## Discussion

The impact of BMI, particularly obesity, on the prognosis of colon carcinoma after surgery is discussed controversially. In the present study, in addition to BMI, several other important prognostic factors, such as age, ASA classification, emergency surgery, tumor location, and tumor stage, were identified. However, the favorable influence of obesity on disease-free and overall survival was also mathematically confirmed in the multivariate analyses.

Yang et al. confirmed that obesity is an independent prognostic factor for colon cancer patients and investigated the molecular mechanisms that might lead to this conclusion. One finding was a downregulation of miR-210 [20]. MicroRNAs (miRNAs) are small noncoding RNAs that are mainly implicated in posttranscriptional gene silencing by interacting with the untranslated region of transcription [21]. In cancer metabolism, hypoxia may promote cancer invasiveness, aggressiveness, and treatment resistance. In obese patients, miR-210 is inhibited, leading to suppression of hypoxia related pathways and preventing cancer progression [20].

In contrast, the study by Gan et al. found an association between obesity and histological tumor budding at the invasive margin of the tumor. In their study cohort, obesity was proven to be an independent risk factor for advanced tumor budding [22], which is a risk factor for poorer survival [23]. Petrelli et al. found in a systematic review and meta-analysis that obese colorectal cancer patients have a worse prognosis than normal-weight patients, as do patients with breast, prostate, and gastroesophageal cancer. In contrast, patients with obesity and lung cancer, renal cell carcinoma, or melanoma have better survival than patients without obesity and with the same cancer type [24].

### The obesity paradox

However, our results may also indicate a phenomenon already found in other diseases, such as cardiovascular disease [25], osteoporosis [26], or end-stage renal disease [27], and is frequently called the “obesity paradox”. This phenomenon indicates that obesity, which is actually a well-known risk factor for several diseases, including colon cancer, may also result in improved survival rates and reduced mortality rates [20]. The results of this study may even suggest that once colon cancer has manifested, the prognosis could be improved by being overweight or even by being obese.

Some recent studies on the topic of the obesity paradox have tried to find the cause for this phenomenon in methodology by the following considerations. These methodological explanations include, in particular, the crudeness of BMI as an obesity measure, reverse causality, confounding, collider-stratification bias, and detection bias [28, 29].

#### Crudeness of BMI

BMI is the most commonly used method for detecting underweight or overweight. BMI has high specificity but low sensitivity, which can result in underdetection of obesity. This in turn weakens the association between obesity and disease. As BMI does not distinguish between muscle and fat [30], it does not provide adequate information on body composition. However, this information is of high relevance because cancer patients in all BMI groups showed high variability in fat and muscle distribution, and increased visceral fat mass and reduced muscle mass have been linked to lower cancer survival [29].

#### Reverse causality

Cancer is known to cause weight changes caused by loss of appetite or increased metabolic demands. Weight loss, especially unintentional weight loss prior to diagnosis, is a common symptom of colon cancer but is associated with advanced tumor stage or more aggressive histology and poor prognosis [30]. When formerly overweight or obese patients may be normal weight at diagnosis, this may falsify correlations between BMI and mortality [31] because obese or overweight patients move into the normal weight group, and their unfavorable prognosis then becomes apparent in the normal weight group.

It has been described that weight loss sometimes begins as early as 6 months before cancer diagnosis, and the effects on lipid metabolism may even begin as early as 2 years before diagnosis [32]. Campbell et al. found that CRC patients who experienced weight loss (more than 10 pounds) both before and after diagnosis had a significantly higher risk of death [33]. The timing of BMI determination may also be highly relevant. Wu et al., for example, found that in CRC patients, a high BMI before diagnosis was associated with a poorer prognosis, while patients with a high BMI after treatment had a better prognosis [34].

#### Confounding

Confounding in the case of the obesity paradox can be caused, for example, by unmeasured or inadequately measured variables that may influence the patient’s health status or weight, such as smoking, socioeconomic status, physical activity, and diet. These data are sometimes difficult to record, especially in retrospective analyses [29].

#### Collider stratification bias

Another possibility that could be used to explain the obesity paradox is a particular form of selection bias, the so-called collider stratification bias [31]. According to Park et al., selection bias occurs when a study population is stratified by a variable that is influenced by exposure and outcome. This can create a false association or even an inverse association between exposure and outcome. The authors argue that obesity may be linked to cancer incidence and that cancer in turn may be associated with an increased risk of death. The same is true for smoking. Obese people may have developed cancer either from obesity or from smoking, while nonobese people may have developed cancer because of smoking. This means that nonobese cancer patients are more likely to be smokers than obese cancer patients. This would make smoking a stronger risk factor for cancer incidence and cancer mortality, suggesting that obese cancer patients have a lower risk of death than obese people [27, 29, 35].

#### Detection bias

The detection bias is based on the fact that two diagnoses occur together. If a patient is diagnosed with one disease, subsequent diagnostics may accidently discover other diseases, such as cancer, that have previously gone undetected. Often, these asymptomatic cancers are at an early stage. Consequently, it is to be expected that the diagnosis of early stages of disease also results in a better overall prognosis, which in turn can be used as a possible explanation for the obesity paradox [31]. In our study, however, this explanation was ruled out by the multivariate analysis, including the stage of disease.

### Underweight patients

In this study, the group of underweight patients compared to the group of normal weight patients showed a nonsignificantly better outcome in the univariate analyses but a rather worse prognosis in the multivariate analyses. These findings might be limited to the small sample size of 13 patients. Sinicrope et al. found an association between underweight colon cancer patients and shorter time to recurrence and disease-free survival [36]. In general surgery, underweight patients were found to be most at risk of major postoperative complications, including long-term mortality [37]. Among colon cancer patients who received adjuvant treatment, a greater overall mortality, especially a twofold higher risk of noncolon cancer deaths, was found in underweight patients [38]. According to Kasi et al., underweight might be a surrogate marker for an advanced or aggressive nature of disease that is combined with less tolerated treatment [39]. Underweight may not only indicate increased metabolic activity due to more aggressive tumor biology but also reflect a lack of nutritional reserves [40].

The strengths of this study are the prospective data collection and the long follow-up. Weaknesses include the fact that BMI was only collected once directly before surgery, and we were not able to include weight changes in our statistical analyses. Shahjehan et al. demonstrated that this is particularly interesting. They found that the decrease in BMI after diagnosis by more than 10% compared to before diagnosis was associated with poorer overall survival in multivariate analysis [41].

## Conclusion

In conclusion, our study showed that in 694 patients with colon carcinoma treated with CME, the group of overweight and even obese patients did not have worse outcomes than normal weight patients. The favorable influence of obesity on disease-free and overall survival was even mathematically confirmed in the multivariate analyses. However, this must be interpreted with caution, as reverse causality, confounding, stratification, and detection biases were not taken into account in the analysis. These observations correspond to the phenomenon of the obesity paradox.
